# Design and Performance Validation of a High-Voltage Controller for MFC Piezoelectric Sensing and Actuation

**DOI:** 10.3390/s26165064

**Published:** 2026-08-10

**Authors:** Qiong Zhu, Jinhao Qiu, Hong Lei

**Affiliations:** 1 State Key Laboratory of Mechanics and Control for Aerospace Structures, Nanjing University of Aeronautics and Astronautics, Nanjing 210016, China; june@nuaa.edu.cn; 2 College of Aeronautic, Nanjing University of Aeronautics and Astronautics, Nanjing 210016, China; leihong@nuaa.edu.cn

**Keywords:** Macro Fiber Composite (MFC), piezoelectric sensing, capacitive load, active vibration control

## Abstract

In aerospace applications, structural vibration can cause fatigue accumulation and shorten the service life of aircraft. This makes vibration suppression based on Macro Fiber Composite (MFC) piezoelectric composites an important research topic. Considering the asymmetric high-voltage operating range of the M-8557-P1 MFC from −500 V to 1500 V and its capacitive impedance characteristics within the 1000 Hz operating frequency band, this paper designs a laboratory prototype of a high-voltage driver. The prototype adopts a voltage–current dual closed-loop structure and a current-tracking PWM control strategy. Under the tested laboratory conditions, the prototype exhibited a relatively fast transient response and a certain dynamic driving capability for capacitive loads. Based on the laboratory prototype, an auxiliary signal-conditioning module and a digital control module equipped with an active control algorithm were further developed. These modules were integrated with the laboratory prototype to form a high-voltage closed-loop control system for MFC piezoelectric sensing and actuation. Ground laboratory tests were conducted on a high-aspect-ratio unmanned aerial vehicle wing. The experimental results show that, when the dominant vibration frequency is approximately 3.6 Hz, the response converges to a steady state within 4.77 s after control is applied. In the steady state, the root-mean-square displacement decreases from 15.57 mm to 4.28 mm, corresponding to a reduction of 72.52%. This result demonstrates the effectiveness of the active vibration control system under this representative application scenario.

## 1. Introduction

In aerospace engineering, structural vibration remains a persistent issue, as aircraft often operate under aerodynamic loading, engine excitation, and aeroelastic coupling. Excessive vibration not only undermines the stability of precision equipment such as flight control, navigation, and measurement systems but also accelerates fatigue damage accumulation and shortens service life. Therefore, vibration control of aerospace structures has become an important research topic in aerospace engineering. Existing approaches generally fall into passive, semi-active, and active control [1,2]. In essence, they reduce system response and improve overall performance by altering structural dynamics and the transmission paths of vibration energy and acoustic radiation. Passive control is simple and reliable, but its effectiveness is often limited at low frequencies and under varying operating conditions. Semi-active control provides a compromise between power consumption and performance. Active control relies on a closed loop of sensors, controllers, and actuators, applying control forces in real time according to the structural response, thereby achieving greater suppression potential in complex conditions. With the development of smart material technology, active vibration control based on smart actuators has attracted extensive attention due to its fast response, low added mass, and ease of structural integration [3,4].

Among smart materials, piezoelectric materials have been widely used in vibration control systems for beams, plates, shells, and wing-like structures because of their high electromechanical coupling efficiency, wide bandwidth, and rapid response [5]. Research on piezoelectric vibration control mainly follows two routes: active piezoelectric control and piezoelectric shunt damping. The former applies control signals to piezoelectric actuators through external controllers and power-driving circuits to achieve closed-loop suppression; the latter modifies the electrical boundary conditions of piezoelectric elements through external circuitry to dissipate vibration energy [6]. Compared with conventional piezoceramic patches, MFCs feature a piezoelectric-fiber composite architecture with interdigitated electrodes. This unique structure enables efficient bending and extension for active vibration control, while offering good compliance, surface conformability, and strong actuation and sensing capabilities [7,8]. MFCs can adapt well to curved, lightweight, thin-walled structures, demonstrating strong potential for applications in active vibration control, energy harvesting, and structural health monitoring [9,10].

In the field of vibration control, MFC, as a capacitive actuator with an asymmetric high-voltage operating range from −500 V to 1500 V, requires the driving system to have good transient response capability and dynamic driving capability for capacitive loads. Conventional high-voltage controllers generally consist of separate driving power supplies and controllers, resulting in relatively large system volume and weight, which limits their application in aerospace fields. Therefore, the independent design of drivers that can be integrated with controllers is an important research direction for vibration-suppression control.

At present, mainstream drivers can be mainly classified into two categories according to their operating principles: linear amplifiers and switching-mode drivers. Linear amplifiers have the advantages of high bandwidth, low output ripple, and high control accuracy, making them suitable for applications requiring high signal fidelity. However, they have relatively low efficiency and suffer from severe heat generation under high-power output conditions. Switching-mode drivers have the advantages of high efficiency, high power density, and strong load-driving capability, making them suitable for applications sensitive to volume, weight, and efficiency. Agbossou et al. [11] designed a Class-D amplifier for high-power piezoelectric loads in sonochemical cavitation reactors. The amplifier operates within a frequency range of 10–100 kHz, can deliver nearly 2 kW power to piezoelectric loads, and achieves an efficiency exceeding 95%. Zsurzsan et al. [12] designed a Class-D driver for piezoelectric servo motors. The driver adopts a 200 V DC input, provides an output voltage range of 20–180 V, and has a designed output frequency range of 0–200 Hz, with experimental verification up to 250 Hz. These studies indicate that switching-mode topologies are suitable for improving efficiency and output power. However, switching ripples, electromagnetic interference, and phase characteristics may enter the active control loop, affecting sensing signal quality and the stability margin of the system.

To balance high-voltage output, low noise, and dynamic response, hybrid high-voltage drivers have gradually attracted attention. Pisenti et al. [13] proposed an ultra-low-noise high-voltage piezoelectric driver based on a hybrid architecture combining a flyback switching regulator and high-speed operational amplifiers. The driver can provide an output voltage of approximately 250 V and can be extended to above 1 kV through modification, making it suitable for low-current closed-loop piezoelectric driving. Stiebel and Janocha [14] proposed the concept of hybrid amplifiers for piezoelectric actuators at an early stage, providing ideas for subsequent driver topologies combining linear and switching stages. Zhong et al. [15] further proposed a Class AB-D high-dynamic power amplifier, which combines the linear amplification capability of Class AB and the high-efficiency output capability of Class D. The maximum output voltage is 100 V, and the output power is 20 W. Under a 1 μF capacitive load, both the rise time and fall time are less than 40 μs. Under a 2.2 μF load, the rise time is approximately 60 μs and the fall time is approximately 42 μs.

At the current research stage, most general-purpose power supplies provide symmetric outputs, while dedicated high-voltage drive systems with asymmetric outputs tailored for MFC actuators remain relatively uncommon. Bilgen et al. [16] proposed a lightweight passive solid-state voltage divider composed of diodes and resistors to address the mismatch between the positive and negative allowable voltages of MFC bimorph structures. However, the work focused on voltage balancing rather than the development of a dedicated asymmetric high-voltage power supply for MFCs. Verne et al. [17] designed a three-level NPC high-voltage driver with an output range of −220 V to 800 V and a maximum operating frequency of 200 Hz. The reported experimental validation was conducted with resistive loads, while experimental data for MFCs or capacitive loads were not provided. Krishnan et al. [18] obtained high-voltage DC through voltage multiplication and flyback conversion, and generated high-voltage AC output using H-bridge inversion combined with LC filtering. The reported work was mainly focused on circuit design and MATLAB/Simulink simulation, without presenting experimental results from a hardware prototype. Among commercially available drivers, products from CoreMorrow Ltd. and Smart Material Ltd. are representative examples.

CoreMorrow Ltd.’s E00/E01 modular piezoelectric controller has a relatively high level of commercial maturity [19]. If its upper-computer-based control scheme is used for MFC wing active vibration-suppression experiments, the CoreMorrow E00/E01 chassis and internal backplane, the E18 display and communication module, and the E08 high-voltage power module are typically required. Meanwhile, because the output signal of the sensing MFC has high impedance, weak charge/voltage characteristics, and susceptibility to interference, compatible external MFC signal conditioning, data acquisition, and real-time control units must also be designed. If the closed-loop control process requires independent acquisition of the actual high-voltage output across the actuating MFC, a high-voltage differential or isolated voltage-measurement stage must be added at the output side of the E08 module, and the measured low-voltage equivalent signal must be sent to the upper computer through an external ADC. This measurement stage must satisfy the requirements of high-voltage insulation rating, common-mode voltage range, measurement bandwidth, and safety protection.

This scheme can realize high-voltage MFC driving and provides relatively mature human–machine interaction and communication functions. However, its system architecture is mainly oriented toward general-purpose benchtop testing, and the module interfaces, communication protocols, and command timing are constrained by the existing product system. When it is interfaced with a self-developed controller or a real-time vibration-control algorithm, secondary development is still required, including communication-protocol adaptation, command-timing design, data exchange, and reconstruction of the external control unit. In addition, the multi-stage communication chain may introduce closed-loop delay and timing uncertainty, and its influence on the stability and real-time performance of MFC active vibration control still requires further verification. Furthermore, the chassis-based modular structure and multiple external functional units increase the system volume, connection links, and integration complexity. This is not conducive to the compact integration of MFC sensing, acquisition, control, and high-voltage actuation functions, and also increases the difficulty of further engineering adaptation for aerospace applications.

Smart Material Corp.’s microHVA-2 is a miniature high-voltage driver board designed for MFC P1-type products [20]. It features a compact size and is suitable for integrated design in aerospace applications. It provides two adjustable outputs of 0–2000 V and one fixed +500 V bias output. However, under a 100 nF MFC load, the maximum sinusoidal frequency corresponding to a 2000 V voltage swing is 2 Hz, while the maximum sinusoidal frequency corresponding to a 1000 V voltage swing is 4 Hz, indicating that its dynamic output capability is limited under 100 nF capacitive loads and high-voltage swing conditions.

Liu et al. [21] integrated the acquisition system, controller, and driver for MFC applications and applied the system to a wing for vibration-suppression experiments. The transient response showed a rise time of 0.7 ms. The driver was adapted from a commercial 0–2000 V DC-output driver supplied by CoreMorrow. However, the asymmetric high-voltage driving requirements of MFC actuators still require dedicated driver design.

This paper designs a laboratory proof-of-concept prototype of an asymmetric high-voltage driver for MFC capacitive loads, targeting the integrated application of MFC sensing and actuation in active vibration control of flexible wings. The prototype adopts a voltage–current dual closed-loop H-bridge PWM topology. Based on this topology, modules including MFC signal acquisition, conditioning, digital control, and isolation protection were designed and fabricated, forming a laboratory hardware platform for wing active vibration-suppression experiments. The transient response and capacitive-load dynamic output characteristics of the prototype are verified through no-load step tests, frequency response tests under different capacitive loads, output ripple tests, and temperature rise tests. In addition, a closed-loop vibration-suppression experiment on a wing was conducted under specific laboratory conditions to verify the system functionality. As a proof-of-concept prototype study, this work may provide a potential approach for future integration and optimization of MFC sensing, control, and actuation systems.

The remainder of this paper is organized as follows. Section 2 presents the overall architecture of the controller, the high-voltage power circuit, and the signal acquisition and digital control units, and establishes the voltage–current dual closed-loop control model. Section 3 describes the prototype and test platform, and analyzes the step response, capacitive-load frequency response, output ripple, and thermal characteristics of the driver. Section 4 validates the closed-loop control functionality of the developed prototype system under single-frequency excitation through active vibration-suppression experiments on a flexible wing. Section 5 concludes the paper.

## 2. Overall Design of MFC High-Voltage Control System

**Figure 1:**
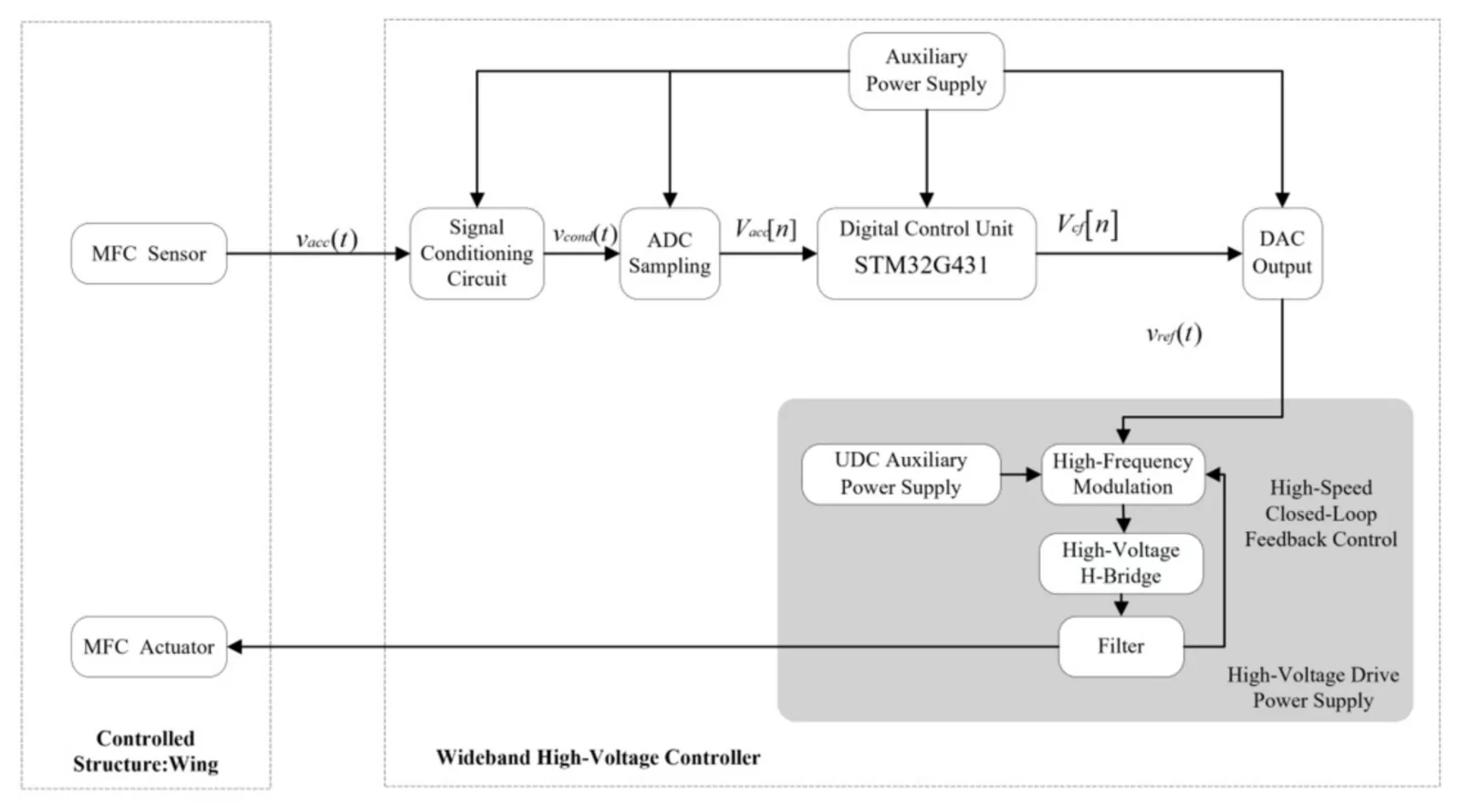
Hierarchical architecture of the high-voltage closed-loop control system for MFC piezoelectric actuation. The system integrates the MFC sensor and actuator with signal conditioning, analog-to-digital converter (ADC) sampling, a digital control unit (STM32G431), digital-to-analog converter (DAC) output, and a high-voltage drive stage with high-speed closed-loop feedback.

MFC actuators primarily rely on the inverse piezoelectric effect. Their operating voltage range spans from −500 V to 1500 V, exhibiting pronounced asymmetric high-voltage characteristics and significant capacitive behavior below 1 kHz. Compared with previous MFC-oriented asymmetric high-voltage drivers, this study places more emphasis on output-voltage retention and dynamic response over the target operating frequency range up to 1000 Hz. To satisfy these drive requirements, this paper presents a high-voltage closed-loop control system, as illustrated in Figure 1, forming a complete feedback loop that includes sensing, data acquisition, control and actuation.

Figure 1 further details the hierarchical system architecture: on the left, the controlled structure with the MFC sensor and actuator is shown, where the sensor detects structural vibrations and the actuator applies active control to suppress them. The upper-middle block represents the digital control module, integrating signal conditioning, ADC sampling, and an STM32G431 controller, which processes sensor data, executes the active-control algorithms, and generates low-frequency reference signals. The lower shaded block corresponds to the high-frequency, high-voltage driving stage, comprising high-frequency modulation, a high-voltage H-bridge, and a filter network, whose primary role is to convert the low-frequency reference signals into high-voltage drive signals suitable for the MFC actuator. This layered architecture effectively decouples low-frequency control from high-voltage power execution, enhancing system integration and implementation flexibility. Detailed analysis of the capacitive load characteristics and four-quadrant operation is provided in Section 2.2.

### 2.1. Design of the High-Voltage Dual-Loop Control Architecture and Small-Signal Model

The high-voltage driver for the MFC capacitive load consists of a full-bridge inverter power stage and a voltage–current dual-loop PWM controller. In the developed prototype, the high-voltage direct-current (DC) bus was set to 1000 V. The power stage converts the unidirectional DC-bus voltage into a bipolar high-voltage actuation signal, while the PWM controller determines the switching states of the power devices according to the voltage and current feedback signals.

For convenience of analysis, $C_{Load}$ denotes the fixed output-filter capacitance, whereas $C_{eq}$ denotes the equivalent capacitance of the MFC actuator or, in the capacitor-loading tests, the externally connected capacitor used to emulate the MFC load. The total equivalent output capacitance at the output terminal is defined as

(1) $$C_{o}=C_{Load}+C_{eq}$$

Unless otherwise stated, the capacitance values reported in the loading tests refer to $C_{eq}$. In the averaged model, the bridge output drives the output terminal through $L_{f}$, with $C_{o}$ representing the total output-side capacitance. The four switches $Q_{1}∼Q_{4}$ together form the high-frequency modulation stage of the high-voltage driver, which generates the switched drive signal for the output network. The output filter inductor $L_{f}$ and the filter capacitor $C_{Load}$ form the high-frequency demodulation network, which suppresses switching components and reconstructs the target high-voltage waveform at the load terminal. In the modulation stage, $Q_{1}$ and $Q_{2}$ operate in current-modulation mode and are mainly responsible for high-frequency current injection, whereas $Q_{3}$ and $Q_{4}$ operate in voltage-modulation mode and are mainly responsible for output-voltage polarity control.

To balance output accuracy, dynamic response, and capacitive-load drive stability, a dual-loop PWM drive controller is introduced. The architecture integrates an outer voltage loop and an inner current loop, so that output-voltage tracking and capacitive-load current regulation can be coordinated through polarity switching and high-frequency PWM modulation.

The outer voltage loop generates the current reference from the voltage-tracking error, thereby maintaining steady-state output-voltage accuracy. The inner current loop regulates the output current according to the current error and participates in the charging and discharging process of the capacitive load. This control arrangement is used to meet the asymmetric AC-excitation requirements of MFC capacitive loads.

The outer loop takes the output voltage $U_{o}$ as the control variable and obtains the voltage error $e_{v}(t)$ by comparing the reference voltage $V_{ref}$ with the feedback voltage.

(2) $$e_{v}\left(t\right)=V_{ref}\left(t\right)-k_{v}U_{o}\left(t\right)$$

where $k_{v}$ is the voltage-sampling scaling factor. The voltage error is processed by the voltage-loop PI regulator $PI_{v}$ to generate the current reference $w_{v}(t)$.

(3) $$w_{v}\left(t\right)=PI_{v}\left\{e_{v}\left(t\right)\right\}$$

The inner loop takes the output current $I_{o}(t)$ as the control variable and obtains the current error $e_{i}(t)$ by comparing the current reference $w_{v}(t)$ with the sampled output current.

(4) $$e_{i}\left(t\right)=w_{v}\left(t\right)-k_{i}I_{o}\left(t\right)$$

The current error is then processed by the current-loop PI regulator $PI_{i}$ to generate the current modulation variable $w_{i}(t)$.

(5) $$w_{i}\left(t\right)=PI_{i}\left\{e_{i}\left(t\right)\right\}$$

Here, $PI_{v}$ represents the outer voltage-loop PI regulator, which generates the current reference $w_{v}(t)$ from the voltage error $e_{v}(t)$. $PI_{i}$ represents the inner current-loop PI regulator, which generates the current modulation variable $w_{i}(t)$ from the current error $e_{i}(t)$. Both regulators adopt a proportional-integral form in the Laplace domain,

(6) $$PI_{v}\left(s\right)=K_{pv}\left(1+\frac{K_{iv}}{s}\right)$$

(7) $$PI_{i}\left(s\right)=K_{pi}\left(1+\frac{K_{ii}}{s}\right)$$

where $K_{pv}$ and $K_{iv}$ are the proportional and integral coefficients of the voltage loop, respectively, and $K_{pi}$ and $K_{ii}$ are the proportional and integral coefficients of the current loop, respectively.

To characterize the output polarity control achieved by $Q_{3}$ and $Q_{4}$, a polarity function $s\left(t\right)$ is introduced:

(8) $$s\left(t\right)=\left\{\begin{array}{l} +1,V_{ref}\left(t\right)\geq 0,\\ -1,V_{ref}\left(t\right)<0. \end{array}\right\}$$

where $s\left(t\right)=+1$ and $s\left(t\right)=-1$ correspond to positive and negative bridge-output polarity, respectively. The current modulation variable $w_{i}(t)$ is compared with the high-frequency triangular carrier Tri to generate two PWM signals, *PWM1* and *PWM2*, which are then mapped by the logic distribution network to the gate-drive signals of $Q_{1}$ and $Q_{2}$.

Let $d\left(t\right)$ denote the equivalent duty ratio of the high-frequency PWM modulator within one switching period, where $0\leq d\left(t\right)\leq 1$. The average bridge-leg voltage can be expressed as

(9) $$u_{ab}\left(t\right)=s\left(t\right)d\left(t\right)U_{DC}$$

where $u_{ab}\left(t\right)$ denotes the average bridge-leg voltage applied to the output filter, and $U_{DC}$ is the DC-bus voltage. The signed normalized modulation variable $m\left(t\right)$ is further defined as

(10) $$m\left(t\right)=s\left(t\right)d\left(t\right),\;-1\leq m\left(t\right)\leq 1$$

Then, Equation (9) can be rewritten as

(11) $$u_{ab}\left(t\right)=m\left(t\right)U_{DC}$$

Equation (11) shows that, in the average sense, the power stage can be equivalent to a modulation link that takes the signed normalized modulation variable as the control input and the average bridge-leg output voltage as the output. The polarity-control branch determines the sign of the output voltage, while the high-frequency PWM modulation branch determines how the output amplitude varies. Together, they unify polarity switching and energy modulation.

The bridge output is applied to the total equivalent output capacitance $C_{o}$ through the output filter inductor $L_{f}$. Neglecting high-frequency switching ripple, the averaged dynamics of the power stage and output filter can be written as

(12) $$L_{f}\frac{dI_{o}\left(t\right)}{dt}=u_{ab}\left(t\right)-U_{o}\left(t\right)-I_{o}\left(t\right)r$$

(13) $$C_{o}\frac{dU_{o}\left(t\right)}{dt}=I_{o}\left(t\right)$$

Here, Io denotes the output-filter inductor current in the averaged power-stage model and is the current regulated by the inner current loop. Because $C_{o}$ includes both $C_{Load}$ and $C_{eq}$, Io represents the total capacitive current at the output terminal in the ideal averaged model. The equivalent damping resistance, r, of the output loop is used to represent the combined effects of winding resistance, device conduction loss, and parasitic damping. Combining Equations (11)–(13) yields the second-order dynamic equation for the output voltage.

(14) $$L_{f}C_{o}\frac{d^{2}U_{o}\left(t\right)}{dt^{2}}+rC_{o}\frac{dU_{o}\left(t\right)}{dt}+U_{o}\left(t\right)=m\left(t\right)U_{DC}$$

This model shows that the proposed driver forms a closed-loop control path from the low-frequency reference command to the high-voltage output waveform through voltage-error regulation, current-reference generation, PWM modulation, and power-stage energy injection.

For controller-gain selection, the averaged model in Equations (12)–(14) was first rewritten in the small-signal form. Around a steady operating point, the PWM modulator and inverter were represented by the equivalent small-signal gain $K_{inv}$ from the current modulation variable $w_{i}(t)$ to the average bridge-leg voltage $u_{ab}\left(t\right)$. The transfer function from $w_{i}(t)$ to the output voltage $U_{o}\left(t\right)$ is then obtained as

(15) $$G_{u}\left(s\right)=\frac{\Delta U_{o}\left(s\right)}{\Delta w_{i}\left(s\right)}=\frac{K_{inv}}{L_{f}C_{o}s^{2}+rC_{o}s+1}$$

Using $I_{o}(s)=CosU_{o}(s)$, the corresponding transfer function from $w_{i}(t)$ to the output current $I_{o}\left(t\right)$ is

(16) $$G_{i}\left(s\right)=\frac{\Delta I_{o}\left(s\right)}{\Delta w_{i}\left(s\right)}=\frac{C_{o}K_{inv}s}{L_{f}C_{o}s^{2}+rC_{o}s+1}$$

**Figure 2:**
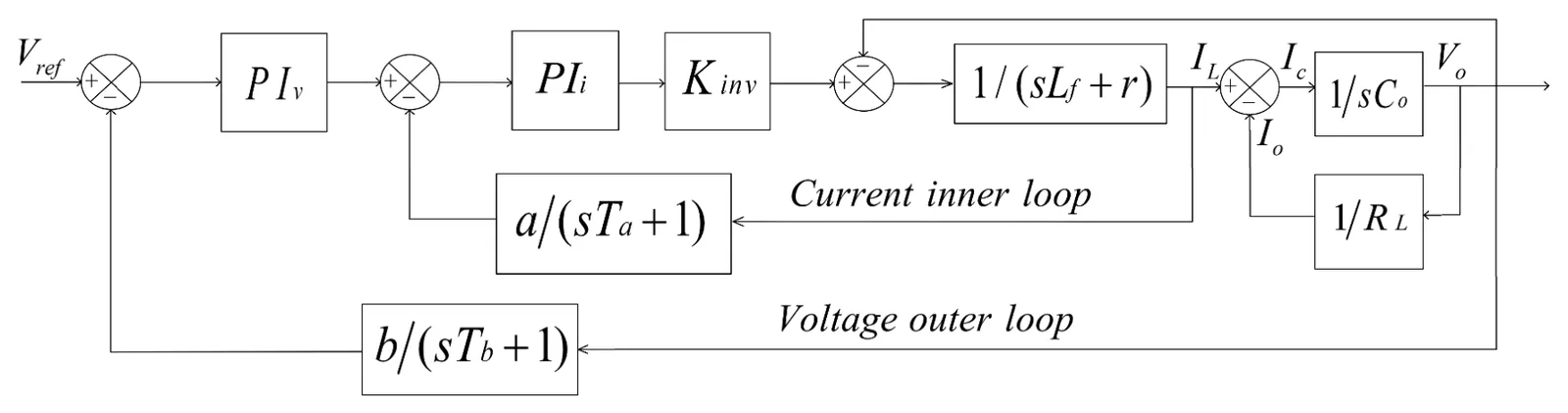
Small-signal block diagram of the voltage–current dual-loop high-voltage driver.

Because the MFC actuator behaves mainly as a capacitive load within the target operating band, the controller was tuned using a standard cascaded-loop-shaping procedure, with the inner current loop tuned before the outer voltage loop. The inner current loop regulates the charge–discharge current of the equivalent output capacitance, whereas the outer voltage loop maintains output-voltage tracking. Bandwidth separation is used to coordinate the dynamic responses of the two loops. Based on the resulting current- and voltage-loop models, the small-signal block diagram of the voltage–current dual-loop high-voltage driver was established, as shown in Figure 2.

According to Figure 2, the complete closed-loop transfer function from the reference voltage $V_{ref}$ to the output voltage $U_{o}$ can be further obtained. In the following expression, $G_{piv}\left(s\right)={PI}_{v}\left(s\right)$ and $G_{pii}\left(s\right)={PI}_{i}\left(s\right)$ denote the voltage-loop PI regulator and the current-loop PI regulator defined in Equations (6) and (7), respectively; $K_{inv}$ is the equivalent inverter gain; $L_{f}$ is the output filter inductance; *r* is the equivalent series resistance of the inductor and power loop; $C_{o}$ is the total equivalent output capacitance defined in Equation (1); $R_{L}$ is the equivalent load resistance; *a* and $T_{a}$ are the current-feedback scaling factor and time constant, respectively; and *b* and $T_{b}$ are the voltage-feedback scaling factor and time constant, respectively. The closed-loop transfer function is therefore obtained as:

(17) $$G(s)=\frac{\frac{K_{\mathrm{inv}}G_{\mathrm{pii}}(s)}{sL_{f}+r}\cdot \frac{1}{\frac{K_{\mathrm{inv}}G_{\mathrm{pii}}(s)}{sL_{f}+r}\cdot \frac{a}{sT_{a}+1}+sC_{o}+\frac{1}{R_{L}}}\cdot G_{\mathrm{piv}}(s)}{1+\frac{b}{sT_{b}+1}\cdot \frac{K_{\mathrm{inv}}G_{\mathrm{pii}}(s)}{sL_{f}+r}\cdot \frac{1}{\frac{K_{\mathrm{inv}}G_{\mathrm{pii}}(s)}{sL_{f}+r}\cdot \frac{a}{sT_{a}+1}+sCo+\frac{1}{R_{L}}}\cdot G_{\mathrm{piv}}(s)}$$

This closed-loop transfer function describes the dynamic relationship between the reference voltage and the output voltage, providing a model basis for analyzing the frequency response, stability margin, and influence of parameter variations.

To further verify the stability of the designed voltage–current dual-loop control structure, the frequency response of the corresponding loop-gain model was analyzed based on the small-signal model described above.

In the complete loop model, the current- and voltage-feedback dynamics were represented by $a/\left({sT}_{a}+1\right)$ and $b/\left({sT}_{b}+1\right)$, respectively, whose low-frequency gains correspond to the current- and voltage-sampling scaling factors introduced in Equations (4) and (2), respectively. The PI gains were further refined experimentally by checking the step response, output-voltage ripple, and capacitive-load stability. During this refinement, the phase margin was required to remain above 45°, the gain margin was kept sufficiently large, and the step response was not allowed to exhibit sustained oscillation or excessive overshoot. Using $K_{inv}=25$, $L_{f}=1\mathrm{mH}$, $C_{o}=C_{Load}=0.94\;\mu F$, and $R_{L}=100\Omega$, the final gains were selected as $K_{pv}=0.88$, $K_{iv}=0.02$, $K_{pi}=1.2$, $K_{ii}=0.6$, a=0.5, $T_{a}=20\;\mu s$, b=0.001, and $T_{b}=20\;\mu s$. With these parameters, the Bode analysis gave a phase margin of 47.1°, a gain margin greater than 60 dB, and a gain crossover frequency $f_{c}$ of approximately 2.9 kHz. These results indicate that the designed voltage–current dual-loop control structure provides sufficient stability margin.

The generated PWM1 and PWM2 signals are mapped to the corresponding gate-drive pulses of the high-voltage H-bridge after logic decoding. In this modulation scheme, the polarity-control branch determines the sign of the bridge output, whereas the high-frequency PWM branch regulates the injected energy within each switching period. Therefore, bipolar high-voltage excitation can be reconstructed from a unidirectional DC-bus supply through the combined action of polarity switching and high-frequency current modulation. As the modulation-wave amplitude varies, the pulse widths of *PWM1* and *PWM2* adjust accordingly, thereby altering the duty ratio of $Q_{1}$ and $Q_{2}$. Consequently, the proposed modulation method maps the information in the low-frequency reference waveform onto high-frequency switching pulses and recovers the target output waveform through the post-stage filter network.

The essence of the above control scheme is to adjust the average injected energy through $Q_{1}$ and $Q_{2}$ within each carrier period, while using $Q_{3}$ and $Q_{4}$ to complete output polarity switching, thereby reconstructing a bipolar high-voltage waveform under a unidirectional DC-bus supply. Since two equivalent modulation events occur within one carrier period when symmetric positive and negative triangular carriers are used, the equivalent modulation frequency is $f_{eq}=2f_{s}$, where $f_{s}$ is the triangular-carrier frequency. For target modulation waves in the 0–1 kHz vibration-suppression scenario, the carrier frequency is typically chosen to be no lower than 20 kHz to ensure good modulation–demodulation performance. A carrier frequency of 20 kHz was used in this study.

In summary, the voltage–current dual-loop controller provides the control basis for coordinated regulation of output voltage and output current, while the bipolar frequency-doubled PWM strategy provides the corresponding high-frequency modulation implementation. Existing MFC high-voltage driving studies have demonstrated the feasibility of asymmetric high-voltage actuation and compact system integration. Compared with such voltage-drive-oriented schemes, the present architecture introduces an inner current loop to coordinate the charging–discharging process of the capacitive load with high-voltage output tracking. Since the MFC actuator behaves as a capacitive load with an inherent voltage–current phase difference, its four-quadrant operating characteristics must be further analyzed to ensure stable drive performance [22].

### 2.2. Electrical Characteristics and Four-Quadrant Operation of the MFC Capacitive Load

The dual-loop control and high-frequency modulation mechanisms described above explain, from a control-structure perspective, how the driver coordinates the output voltage and output current. Since the MFC actuator exhibits capacitive electrical behavior, a phase difference usually exists between output voltage and output current, and the drive process involves charge–discharge behavior and bidirectional energy exchange. Therefore, the electrical characteristics of the MFC load are analyzed below to clarify the operating requirements under capacitive-load conditions.

Within the target operating frequency range below 1000 Hz, the MFC actuator mainly exhibits capacitive load characteristics. In the driver-level analysis, the MFC actuator was represented by an equivalent capacitance, and the capacitance of a single M-8557-P1 actuator was taken as 12.84 nF according to reported M-8557-P1 [23]. Therefore, an equivalent capacitive-load model was used in the driver-level analysis and tests. Under this condition, a phase difference usually exists between the output voltage and output current during driving. The proposed driver combines voltage-polarity switching with high-frequency current modulation to drive the capacitive load.

In the power-stage averaged model and the closed-loop transfer function, $C_{o}=C_{Load}+C_{eq}$ represents the total equivalent output capacitance, and $I_{o}$ denotes the output-filter inductor current. The current drawn by the MFC actuator or the external capacitive load is denoted by $I_{c}$. In the capacitor-loading tests, $C_{eq}$ is the externally connected test capacitor.

(18) $$I_{c}\left(t\right)=C_{eq}\frac{dU_{o}\left(t\right)}{dt}$$

Equation (18) indicates that $I_{c}$ is determined by the rate of change of $U_{o}$ and $C_{eq}$. When $U_{o}$ remains constant, $I_{c}$ tends to zero; when $U_{o}$ changes rapidly, the charge–discharge current increases accordingly. Therefore, for MFC driving, the high-voltage output capability should be considered together with the charge–discharge current demand of the capacitive load under high-frequency operation.

Under ideal capacitive loading, the output current $I_{c}$ leads the output voltage $U_{o}$ by 90 degrees.

Based on the above characteristics, the system sequentially experiences four different voltage–current combinations within one operating period, namely

(19) $$\begin{array}{l} U_{o}>0,I_{c}>0\\ \;U_{o}>0,I_{c}<0\\ U_{o}<0,I_{c}<0\\ U_{o}<0,I_{c}>0 \end{array}$$

Equation (19) indicates that the driver must simultaneously handle both same- and opposite-sign combinations of output voltage and current under practical operating conditions. This represents the basic requirement for four-quadrant operation and serves as an important indicator of whether a high-voltage driver can effectively adapt to piezoelectric capacitive loads.

The high-frequency high-voltage driver proposed in this paper adopts a hybrid modulation topology combining voltage-polarity modulation and high-frequency current modulation. As described in Section 2.1, the four switches $Q_{1}-Q_{4}$ form the high-frequency modulation stage. The $Q_{1}$ and $Q_{2}$ branch provides high-frequency current injection, whereas the $Q_{3}$ and $Q_{4}$ branch determines the output-voltage polarity. This configuration is intended to accommodate the four-quadrant operating requirements of the capacitive MFC load. After passing through the high-frequency demodulation network composed of the output filter inductor $L_{f}$ and the filter capacitor $C_{Load}$, the target high-voltage waveform is recovered at the load terminals.

This filter network both demodulates high-frequency pulses and establishes a dynamic energy-exchange channel between the capacitive load and the driver. For a capacitive load, the stored energy can be expressed as ${W=\frac{1}{2}C_{eq}U_{o}}^{2}$, where W denotes the electric-field energy stored in the MFC actuator or in the external test capacitor represented by $C_{eq}$.

The electric-field energy in the load varies with the output voltage. The capacitive-load characteristics of the MFC actuator inherently involve a phase difference between the output voltage $U_{o}$ and output current $I_{o}$, as well as bidirectional energy exchange. Thus, the proposed control architecture provides a design basis for accommodating the high-voltage and capacitive-load operating requirements described above. The subsequent experiments evaluate its output behavior under the stated laboratory conditions.

### 2.3. Output Filter Design and Parameter Selection of MFC Piezoelectric Actuation

The H-bridge output of a high-voltage driver is essentially a high-frequency modulated pulse train and cannot be applied directly as the target drive voltage required by the actuator. Therefore, an output filter network, consisting of a filter inductor $L_{f}$ and a filter capacitor $C_{Load}$, is required at the power-stage output to suppress high-frequency carrier components and recover the target high-voltage waveform. For the driver designed in this paper, the filter network not only demodulates high-frequency pulses but also directly affects the output bandwidth, ripple level, and dynamic response, thereby making its parameter selection critical.

Under the averaged model, the output filter network can be approximated as a second-order low-pass stage from the average bridge output voltage to the output-terminal voltage. When the equivalent damping is small, its natural resonant frequency is primarily determined by $L_{f}$ and the total equivalent output capacitance $C_{o}$. To ensure that the target low-frequency modulation signal passes while high-frequency carrier harmonics are suppressed, the filter parameters should satisfy the following principle: the resonant frequency of the filter network should be higher than the highest frequency of the target modulation signal and lower than the switching-carrier frequency. This ensures good waveform-tracking capability within the target operating band while effectively attenuating high-frequency switching harmonics and reducing output ripple.

The resonant angular frequency of the filter network can be expressed as

(20) $$\omega_{0}=\frac{1}{\sqrt{L_{f}C_{o}}}$$

The corresponding resonant frequency is

(21) $$f_{0}=\frac{1}{2\pi \sqrt{L_{f}C_{o}}}$$

Let the maximum frequency of the target modulation signal be *f*_max_, and let the switching-carrier frequency be *f*_s_; then the filter parameters should satisfy

(22) $$f_{\max}<f_{0}<f_{s}$$

Equation (22) provides the basis for selecting $L_{f}$ and the total equivalent output capacitance $C_{o}$. If the resonant frequency $f_{0}$ is too low, the output filter network may degrade the driver’s high-frequency tracking performance, resulting in reduced effective bandwidth. Conversely, if $f_{0}$ is too high, suppression of high-frequency carrier harmonics will be reduced, increasing output ripple and lowering waveform smoothness. Hence, the design of $L_{f}$ and the total equivalent output capacitance $C_{o}$ should balance dynamic response speed with high-frequency ripple suppression.

Furthermore, within a high-frequency switching subinterval, if the voltage across the inductor remains approximately constant, the inductor current ripple can be approximated as

(23) $$\Delta i_{L}\approx \frac{V_{L}DT_{s}}{L_{f}}$$

where $V_{L}$ is the equivalent voltage across the inductor during the switching subinterval, *D* is the duty ratio, and $T_{s}$ is the switching period. Equation (23) shows that increasing $L_{f}$ reduces the inductor current ripple and improves output smoothness, but an excessively large $L_{f}$ will degrade system response speed. Similarly, increasing $C_{Load}$ strengthens suppression of output-voltage pulsation, while also affecting the filter network’s natural frequency and the system bandwidth.

Since the output-side capacitance in the averaged model is $C_{o}=C_{Load}+C_{eq}$, the parameter analysis was conducted in terms of $L_{f}$ and $C_{o}$. According to the resonant-frequency relationship above, increasing either $L_{f}$ or $C_{o}$ reduces the LC resonant frequency.

A model-based parameter sweep was conducted around the actual parameter values of the prototype. During the sweep, all circuit and controller parameters other than $L_{f}$ and $C_{o}$ were kept constant, while $L_{f}$ and $C_{o}$ were varied independently. The nominal small-signal reference condition was defined as $C_{eq}=\;0$. Under this condition, $C_{o}=C_{Load}=\;0.94\;\mu F$, $L_{f}=1\mathrm{mH}$, r=0.0011Ω, and $R_{L}=100\Omega$.

The swept values of $L_{f}$ were 0.5, 0.75, 1.0, 1.25, 1.5, and 2.0 mH, corresponding to 0.5–2 times the selected filter inductance. The swept values of the output-filter capacitance $C_{Load}$ were 0.47, 0.94, 1.41, 1.88, 2.2, and 3.3 μF.

For each combination of $L_{f}$ and $C_{o}$, the LC resonant frequency was recalculated while the remaining circuit and controller parameters were kept unchanged. Because filter resonance limits the high-frequency voltage-tracking capability of the driver, the relative variation in resonant frequency $f_{o}$ was used to represent the relative variation in bandwidth. A lower $f_{o}$ corresponds to a slower voltage response; therefore, the response-time index was defined as the inverse of the bandwidth index. The ripple index was normalized with respect to variations in $L_{f}$ or output capacitance, because larger filter parameters generally reduce current or voltage ripple.

**Figure 3:**
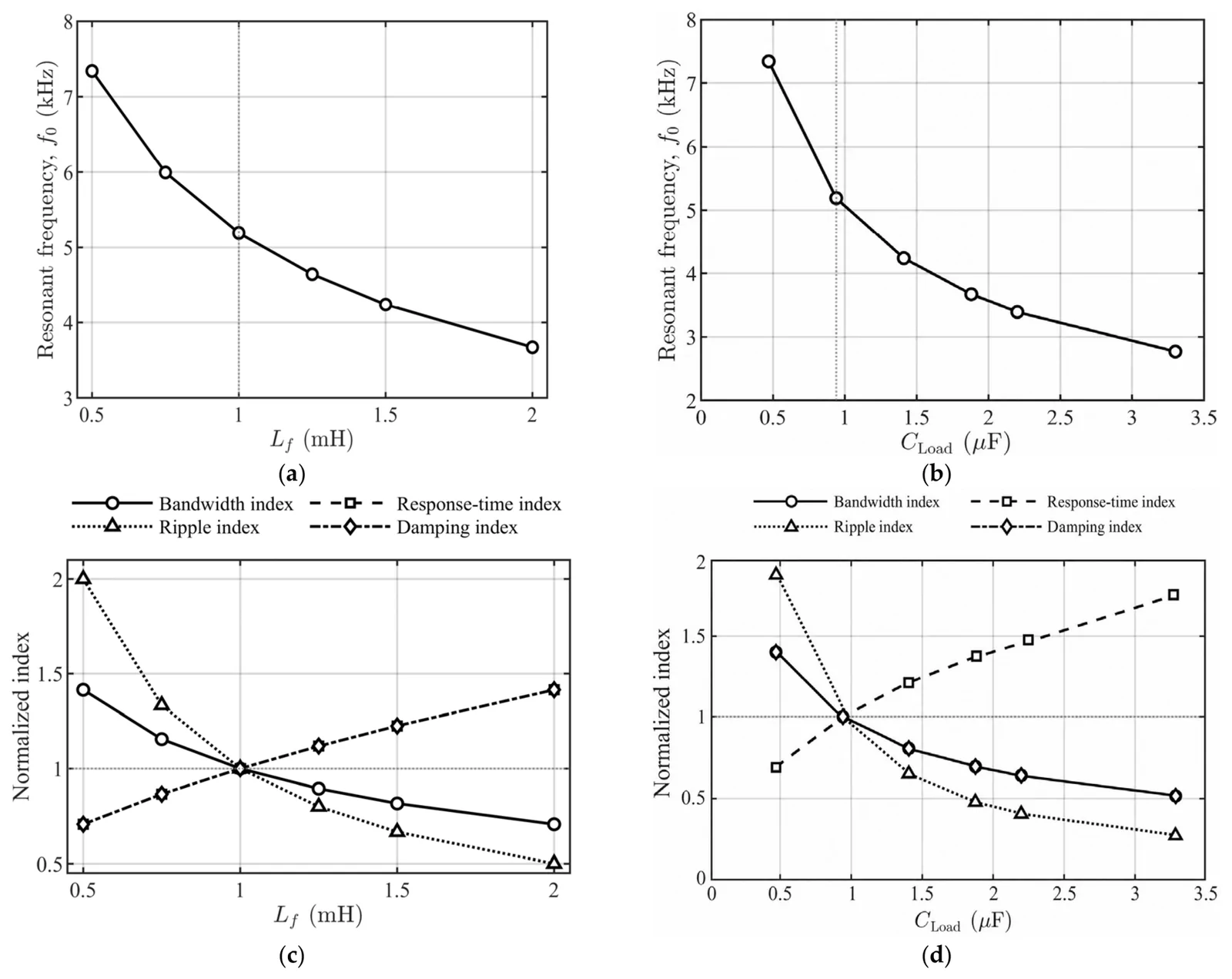
Model-based sensitivity analysis of alternative output-filter configurations with $C_{eq}=0$: (**a**) resonant frequency versus $L_{f}$ with $C_{Load}$ fixed at 0.94 μF. (**b**) Resonant frequency versus $C_{Load}$ with $L_{f}$ fixed at 1 mH. (**c**) Normalized bandwidth, response-time, ripple, and damping indices versus $L_{f}$. (**d**) Normalized bandwidth, response-time, ripple, and damping indices versus $C_{Load}$.

The calculated sensitivity results are shown in Figure 3. Figure 3a,b show that increasing either $L_{f}$ or $C_{Load}$ reduces the LC resonant frequency. Figure 3c,d further show that increasing $L_{f}$ or $C_{Load}$ lowers the normalized bandwidth index and increases the response-time index, while the ripple index decreases with larger filter parameters. The damping index increases with larger $L_{f}$, but decreases as $C_{Load}$ increases. Under the reference condition of $L_{f}=1\mathrm{mH}$ and $C_{eq}=\;0$, $C_{o}=C_{Load}=\;0.94\;\mu F$, the LC resonant frequency is approximately 5.19 kHz and the damping factor is approximately 0.163. These results quantitatively describe the influence of the output-filter inductance and capacitance on the high-frequency voltage-tracking characteristics in the model. Therefore, the output-filter parameters should be selected by considering ripple suppression, bandwidth, damping, and transient response.

Based on the system design, control strategy, and parameter selection described above, the hardware implementations are presented next. The driver performance is then evaluated through step-response, frequency-response, output-ripple, and thermal-behavior tests.

### 2.4. Hardware Implementation and Engineering Protection Considerations

The developed high-voltage actuation system adopts a layered hardware structure consisting of a sampling/control board, an isolation board, and an H-bridge power board. The sampling/control board is responsible for low-voltage command generation and feedback acquisition. A 16-bit high-precision Digital-to-Analog Converter (DAC) generates the control reference, which is buffered and conditioned by an operational amplifier before being sent to the high-voltage modulation stage. A 16-bit Analog-to-Digital Converter (ADC) is used to acquire the MFC terminal-voltage feedback, current-related feedback, and external sensing signals. On the sampling board, the analog and digital ground domains are separated, and local decoupling capacitors are placed close to the ADC, DAC, voltage reference, and power inputs to reduce the influence of digital switching noise and power-supply ripple on the analog measurement path.

The high-voltage power stage is implemented using an H-bridge topology. It receives the high-voltage DC bus input and generates the bipolar high-voltage drive signal required by the MFC actuator through PWM and output filtering. Voltage and current sensing channels are included on the power board to monitor the high-voltage output and the current variation during capacitive-load charging and discharging. These feedback signals are used for the voltage–current dual-loop control and for abnormal-condition detection. For the experimental setup, the prototype includes circuit provisions for overvoltage protection, overcurrent protection, short-circuit protection, current limiting, fuse protection, and residual-charge discharge. Fault interfaces, including the high-voltage fault output (HV_ERR_OUT) and high-current fault output (HC_ERR_OUT), are designed to transmit abnormal-state information to the control board so that the drive command can be limited or shut down when abnormal high-voltage or current conditions are detected.

To reduce the influence of high-voltage PWM switching on the low-voltage sampling and control circuits, an isolation board is inserted between the low-voltage control side and the high-voltage power side. Isolated DC/DC supplies and isolated signal-conditioning circuits are used for power and signal isolation. LC filters, common-mode inductors, and local decoupling capacitors are also arranged at the interface. Together with the separated ground design on the sampling board and the output filter of the H-bridge, these arrangements are used to reduce the influence of high-frequency switching noise propagation through power lines, signal lines, and ground-return paths, thereby improving the stability of feedback acquisition and command transmission in the present experimental platform. Standardized electromagnetic compatibility testing, long-duration operation tests, and thermal reliability evaluation remain necessary in future work to further assess the engineering applicability of the high-voltage driver.

## 3. Performance Test and Analysis of the High-Voltage Driver

Based on the system architecture described above, an experimental platform was constructed to evaluate the output dynamic performance of the high-voltage driver. The experimental platform consisted of an adjustable high-voltage DC power supply (YM200MHYN), a low-voltage regulated power supply (RIGOL), the developed high-voltage driver prototype, a signal generator (Tektronix), a high-voltage differential probe (Tektronix), and a digital oscilloscope (RIGOL). The signal generator provided the reference signal to the driver. The high-voltage output was measured using the differential probe and recorded by the oscilloscope. This section evaluates the step response, amplitude-frequency response under capacitive loading, output-voltage and current waveforms, output ripple, and short-term thermal behavior.

### 3.1. Step Response Characteristic Test

Step response is an important indicator for evaluating the transient response capability of a power supply system. The rise time and fall time respectively reflect the dynamic response speed of the high-voltage driver during voltage build-up and voltage decay. In this section, a square-wave signal with a frequency of 10 Hz and a duty cycle of 50% was generated by a signal generator and used as the control signal. It was applied to the input terminal of the laboratory prototype of the high-voltage driver to test its transient response capability. In this paper, the time required for the output voltage to rise from 10% to 90% of its amplitude is defined as the rise time, while the time required for it to fall from 90% to 10% is defined as the fall time.

Under no-load conditions, the input signal was adjusted so that the driver produced bipolar square-wave outputs with peak amplitudes of 500, 650, 780, and 840 V, corresponding to peak-to-peak voltages of 1.00, 1.30, 1.56, and 1.68 kV, respectively. The measurement results are summarized in Table 1. The experimental results show that the rise time of the output waveform was 18–50 μs and the fall time was 12–30 μs, with the fall time generally shorter than the rise time. The corresponding voltage slew rate was 17.5–70 V/μs. These values characterize the no-load transient response under the stated test conditions.

In addition, the experimental results show that the measured rise time decreased from 50 μs to 18 μs as the step amplitude increased. This phenomenon is related to the switching modulation scheme adopted in the designed driver. In this experiment, the driver was supplied by a fixed 1000 V high-voltage DC bus, and different high-voltage output levels were generated through H-bridge switching and PWM modulation. The higher the target output voltage, the larger the corresponding effective PWM duty cycle and the higher the equivalent average bridge-arm output voltage, allowing the target voltage to be established more rapidly at the output filter. Therefore, under the present test conditions, step signals with larger amplitudes exhibited shorter voltage build-up times. Analysis of the rising and falling edges shows that the system had a short response delay, limited overshoot, and well-damped transient behavior, indicating that the high-voltage driver has good transient response capability.

### 3.2. Frequency Response Analysis of the Symmetric Bipolar High-Voltage Power Driver Prototype

**Figure 4:**
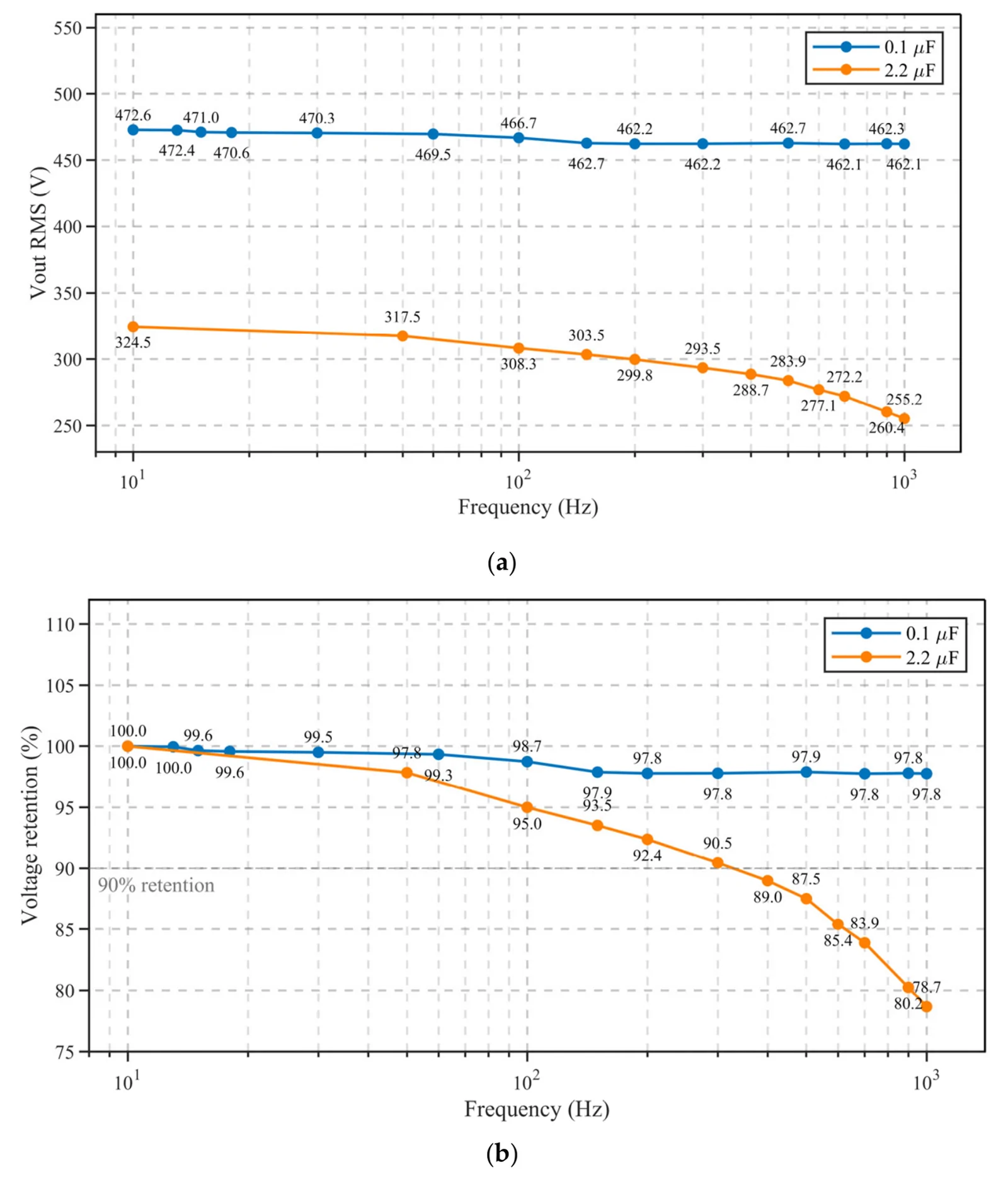
Measured amplitude-frequency response under capacitive loads of $C_{eq}$ = 0.1 μF and 2.2 μF: (**a**) RMS output voltage; (**b**) voltage-retention ratio.

Two representative external capacitors of 0.1 μF and 2.2 μF were used to analyze the amplitude-frequency characteristics under capacitive loading from 10 Hz to 1000 Hz. The 0.1 μF load was used to represent a light capacitive-load condition, whereas the 2.2 μF load was used to represent a heavy capacitive-load condition. For the 0.1 μF load, a 5.3 Vpp sinusoidal input was applied; for the 2.2 μF load, a 3.6 Vpp sinusoidal input was applied. Within each loading test, the input amplitude was kept constant while the input frequency was gradually increased, and the output voltage at each frequency point was recorded. To investigate the effect of different capacitive loads on high-frequency voltage regulation, the test data were evaluated using two indicators: RMS output voltage and voltage retention ratio. The test results are presented in Figure 4a,b. Figure 4a shows the variation in RMS output voltage against frequency under different capacitive loads. Figure 4b provides the corresponding voltage retention ratio curves. For the 0.1 μF capacitive load, the RMS output voltage exhibits only slight fluctuations across the entire test frequency band. The RMS voltage is 472.65 Vrms at 10 Hz and 462.10 Vrms at 1000 Hz. The voltage retention ratio reaches 97.8% at 1000 Hz. When the load capacitance is changed to 2.2 μF, the output voltage decays significantly as frequency increases. The RMS voltage equals 324.46 Vrms at 10 Hz and drops to 255.22 Vrms at 1000 Hz. The corresponding voltage retention ratio decreases to 78.7%. At 1000 Hz, the voltage retention ratio was lower for the 2.2 μF condition than for the 0.1 μF condition. These findings indicate that while the driver possesses excellent wideband tracking capabilities for light loads, the drastic decrease in capacitive reactance at high frequencies under heavy loads challenges the system’s current-supplying capacity, leading to voltage degradation.

### 3.3. Frequency Response Analysis of the Asymmetric Bipolar High-Voltage Power Driver Prototype

**Figure 5:**
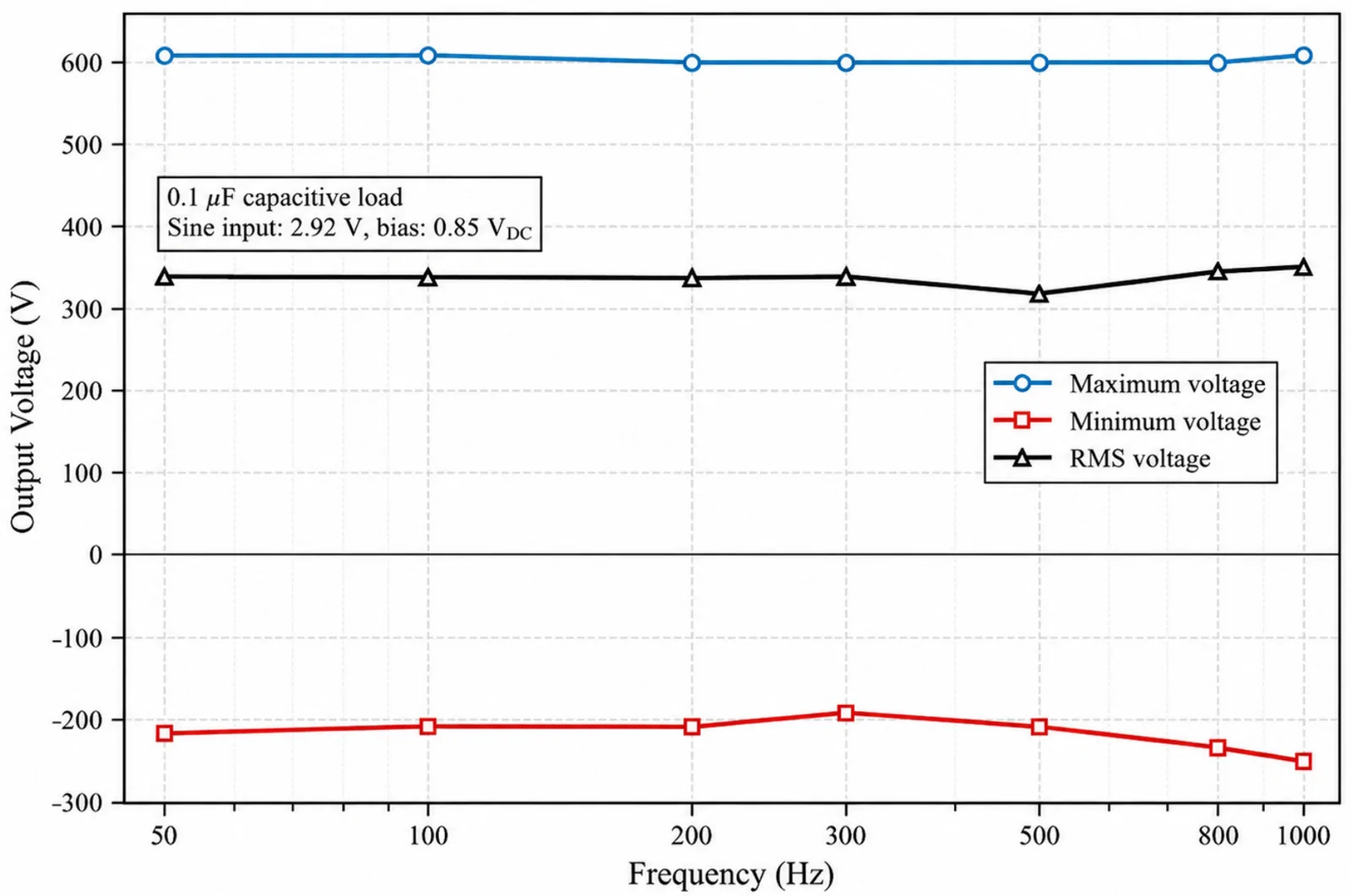
Frequency-dependent output voltage response under asymmetric sinusoidal excitation with a 0.1 μF capacitive load. The input signal was a 2.92 Vpp sinusoidal signal with a 0.85 Vdc bias. The maximum voltage, minimum voltage, and RMS voltage were measured from 50 Hz to 1000 Hz to evaluate the asymmetric output characteristics of the driver under capacitive loading.

Given the pronounced asymmetry of the operating voltage of the MFC actuator, a DC offset was introduced into the control reference signal to better approximate the actual driving requirements and to simulate asymmetric high-voltage output operation. The input signal was set as a sinusoidal signal with a DC offset, where the AC component had a peak-to-peak value of 2.92 Vpp and the DC offset was approximately 0.85 Vdc. At low frequencies, the output voltage level was approximately 338.13 Vrms, with an instantaneous maximum voltage of 608.1 V and a minimum voltage of −216.6 V. Figure 5 presents the output characteristic curves within the operating frequency range up to 1000 Hz. These results indicate that, under the simulated asymmetric voltage condition, the amplifier can maintain a relatively stable output amplitude within the tested frequency band.

### 3.4. Voltage–Current Waveforms Under Capacitive Loading

**Figure 6:**
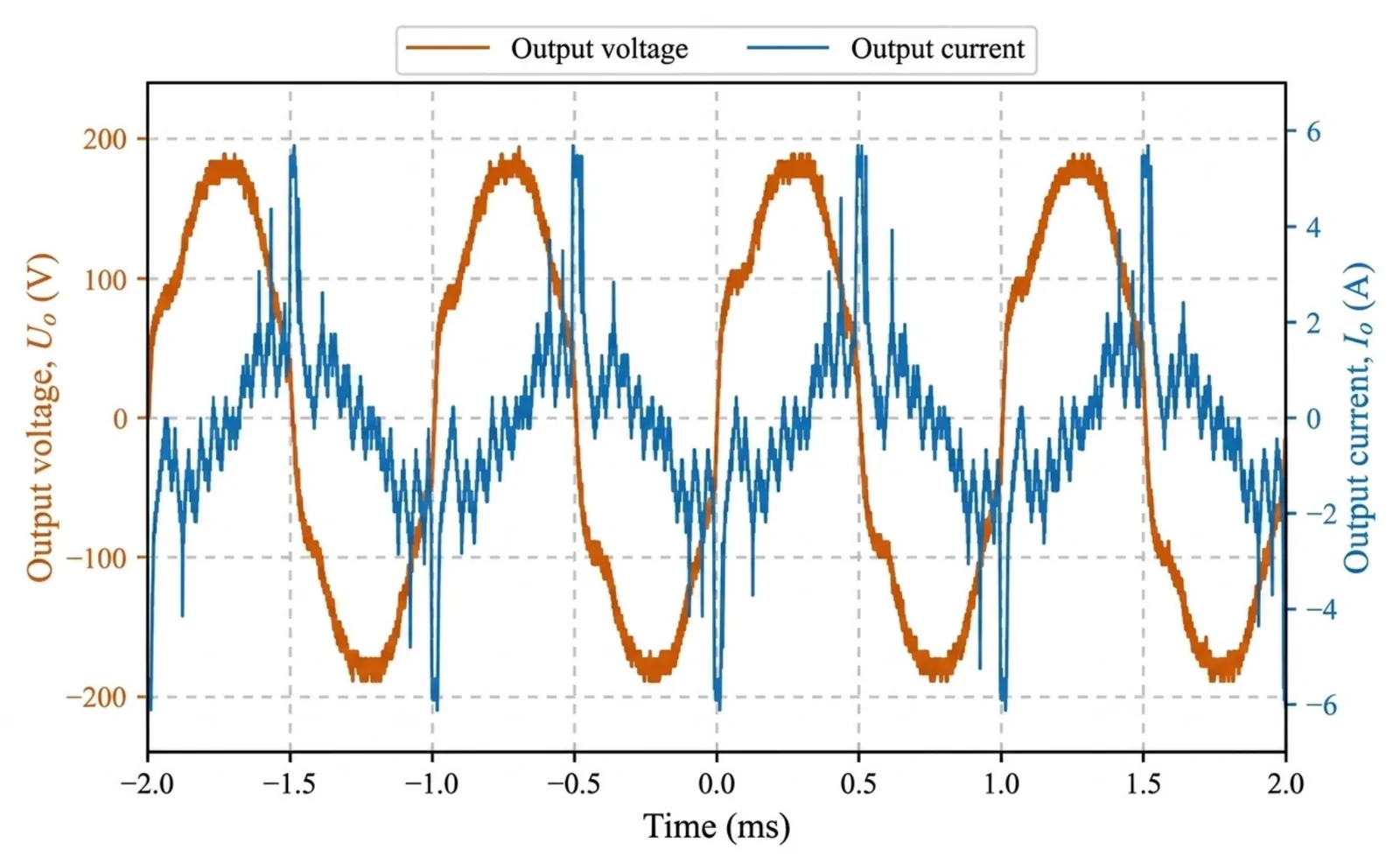
Measured output-voltage and output-filter-inductor-current waveforms under capacitive loading at 1000 Hz. The input reference was a 1.5 Vpp sinusoidal signal with zero DC offset; $C_{Load}=0.94\;\mu F$ and $C_{eq}=0.47\;\mu F$.

To examine the voltage–current waveforms of the driver under capacitive loading, a sinusoidal input signal with a frequency of 1000 Hz, an amplitude of $1.5V_{pp}$ and zero DC offset was applied under the condition of $C_{Load}=0.94\;\mu F$ and $C_{eq}=0.47\;\mu F$. The current probe was placed after the filter inductor $L_{f}$ and before the output capacitor branches to measure the total output-side current $I_{o}$. As shown in Figure 6, the RMS value of the output voltage $U_{o}$ was $134.485V_{\mathrm{rms}}$, and the fundamental RMS value of the total current $I_{o}$ was $1.41A_{\mathrm{rms}}$. The RMS current of the external capacitive-load branch was calculated as $I_{C,\mathrm{rms}}=0.397A_{\mathrm{rms}}$.

Under the passive sign convention, the ideal capacitive branch current $I_{C}$ leads the output voltage $U_{o}$ by $90^{{}^{\circ}}$, consistent with the capacitive voltage–current relationship under this operating condition.

### 3.5. Output Ripple Measurement Under Capacitive Loading

A 0.10 μF capacitive load was used, and the output was set as a 100 Hz sinusoidal high-voltage signal. The output voltage was measured using a high-voltage differential probe and recorded by an oscilloscope as raw CSV data. Under this test condition, the measured output peak-to-peak voltage was 1376.48 V. A 200 μs local segment near the voltage peak was selected, and the low-frequency trend within this segment was removed to extract the high-frequency ripple component. The calculated RMS value of the local ripple was 9.28 V, corresponding to 0.674% of the measured output peak-to-peak voltage. The peak-to-peak value of the local ripple was 70.13 V. These results show that the local high-frequency ripple at the output terminal remained at a relatively low level under the tested capacitive-load condition, indicating that the output filter network provided a certain degree of output-voltage ripple suppression.

### 3.6. Short-Duration Thermal Behavior Under Laboratory Continuous Operation

An infrared thermal-imaging test was conducted to characterize the short-duration thermal behavior of the high-voltage driver under continuous operation. The test was performed in a laboratory environment at an ambient temperature of 25 °C, with heat sinks installed on the power devices. The laboratory prototype was operated continuously for 30 min under the stated test condition. An infrared thermal imager (FOTRIC) was then used to record the surface-temperature distributions of the overall system and the power-stage region.

The maximum surface temperatures of the overall system and the power-stage region with heat sinks were 57.4 °C and 40.2 °C, respectively. Within the 30 min observation period and under the stated laboratory cooling configuration, no obvious abnormal temperature rise was observed. This result is used only to describe the short-duration thermal behavior of the laboratory prototype under the stated test condition.

### 3.7. Parameter Comparison with Other Drivers

At present, most general-purpose high-voltage driving power supplies use symmetric outputs, while power-supply systems specifically designed for the asymmetric high-voltage driving requirements of MFC actuators remain limited. Among commercial products, CoreMorrow E00/E01 modular piezoelectric controllers have a relatively high level of maturity. However, they still need to be used as a complete set together with a chassis, backplane, display and communication module, and high-voltage power module, making them more suitable for general bench testing. For compact integrated scenarios that combine MFC sensing, acquisition, control, and high-voltage driving, interface adaptation, communication timing design, and external control-unit integration still require further work [19].

Smart Material’s microHVA-2 is currently a representative asymmetric board-level high-voltage driving module for MFC applications in the aerospace field [20]. Its published specifications show that, under a 100 nF MFC load, the maximum sinusoidal output frequency is 4 Hz at a voltage swing of 1000 V, and decreases to 2 Hz when the swing increases to 2000 V. By contrast, under the same 100 nF capacitive load, a 1066.50 V swing, and a 10 Hz sinusoidal output, the prototype developed in this paper achieved a measured THD of 1.7328%. Under a relatively high voltage swing and capacitive load, the prototype shows good dynamic driving capability.

For comparison with related work in MFC active vibration suppression, Liu et al. [21] developed an integrated MFC-oriented acquisition system, controller, and driver, and provided power–bandwidth curves under different equivalent capacitive loads. According to their published curve, the output peak-to-peak voltage near 100 Hz under a 470 nF load is approximately 0.40–0.42 kVpp. Under a similar 470 nF external-capacitor condition, an output test was conducted in this study at 100 Hz using a 2.18 Vpp sinusoidal input superimposed on a 0.8 Vdc offset. The measured maximum and minimum output voltages were 499.88 V and −133.30 V, respectively, corresponding to a peak-to-peak voltage of 633.18 Vpp. This result indicates that the present prototype has good dynamic driving capability under the stated capacitive-load condition.

In our previous work, BP neural-network self-tuning was introduced into the driver to enable real-time dynamic adjustment of the digital compensator parameters. In its no-load 0–500 V step test, the rise time was reduced from 1.5 ms for the conventional driver to 0.36 ms. However, this driver and the one developed by Liu’s team were both modified based on commercial DC drivers manufactured by CoreMorrow Ltd. (Harbin, China). In the present study, a bipolar step voltage from −500 V to +500 V was applied under no-load conditions, and a rise time of 50 μs was measured. This result indicates a fast transient response of the developed driver under the stated no-load test condition.

In summary, the laboratory prototype developed in this paper was designed for the asymmetric high-voltage driving requirements of MFC capacitive loads. The measured results show that the laboratory prototype exhibits a fast transient response and good dynamic driving capability under the tested conditions. However, the current system remains a laboratory prototype, and the available results are mainly intended to verify the design concept and experimental feasibility. Further work on engineering integration, environmental adaptability, and long-term reliability is required before practical engineering application can be considered.

## 4. Experiment and Verification of Wing Structural Vibration Control

Research on vibration suppression of high-aspect-ratio wings is critical for the service life of long-duration high-altitude flight [24], and MFC-based active control is recognized as an effective vibration-reduction method [25,26]. The structural validation in this section focuses on the dominant first-bending resonant response of the tested wing.

To suppress low-frequency structural vibration with the piezoelectric actuator, a composite controller was implemented on the proposed high-voltage driving platform. The controller combines feedforward phase compensation with linear active disturbance rejection control (LADRC)-based residual-error correction. The sensing and actuation path includes the sensor, AD7606 sampling module, STM32 controller, AD5761R output module, high-voltage driver, MFC actuator, and the controlled structure. This complete path introduces a non-negligible phase lag. A fixed feedforward phase-compensation coefficient was therefore identified from whole-system experiments and used as the primary phase correction. LADRC was then used to reduce the remaining vibration error.

For the dominant vibration in the 3–5 Hz range, the controller estimates the vibration frequency and phase from the measured input signal. The feedforward control phase is generated by adding a fixed compensation angle to the ideal anti-phase angle of 180°. The commanded phase is given by $\phi_{\mathrm{cmd}}=\phi_{\mathrm{target}}+\phi_{c}$, where $\phi_{\mathrm{cmd}}$ is the commanded control phase, $\phi_{\mathrm{target}}$ is the ideal anti-phase angle, and $\phi_{c}$ is the fixed feedforward phase-compensation angle. In the implemented controller, $\phi_{\mathrm{target}}$ was set to 180.0°, and $\phi_{c}$ was set to 150.0°. The value of $\phi_{c}$ was obtained from repeated whole-system tests. It accounts for the phase shift introduced by signal sampling, digital processing, high-voltage amplification, piezoelectric actuation, and the structural response. Thus, this coefficient is an experimentally identified parameter for the specific driver–actuator–structure system.

The LADRC branch was used as a residual-error feedback corrector after feedforward phase compensation. The discrete sampling period of LADRC was $T_{s}$ = 0.0002 s, corresponding to a 5 kHz control update rate. A third-order extended state observer (ESO) was used, with observer gains $\beta_{1}$ = 84.823, $\beta_{2}$ = 2398.31372, and $\beta_{3}$ = 22,603.5762. The estimated input-channel gain of the controlled plant was $b_{0}$ = 28.0. The controller also included measurement low-pass filtering, anti-saturation compensation, output amplitude limiting, and rate limiting to maintain control stability and protect the high-voltage driver.

In this system, the main phase lag is not caused by the LADRC calculation itself. It mainly comes from measurement filtering, output rate limiting, DAC conversion, high-voltage amplification, MFC actuation, and the structural dynamics. The adopted control structure can therefore be summarized as feedforward phase setting followed by feedback residual correction. The fixed phase-compensation term first compensates for the dominant phase error, and LADRC then corrects the remaining error online. The experimental results show that this strategy provides stable vibration suppression for the low-frequency piezoelectric active control system.

In this study, the high-aspect-ratio wing was used mainly as a test structure for validating the closed-loop active actuation system. The purpose was to examine the performance of the integrated MFC-based active vibration control system on a flexible wing structure. Therefore, Section 4 focuses on the information directly related to the closed-loop experiment, including the placement of the MFC sensor and actuator, the excitation condition, the displacement-measurement method, and the vibration response before and after control. Detailed geometric parameters, material properties, and modal modeling of the wing are not discussed here, as they are not the main focus of this work.

**Figure 7:**
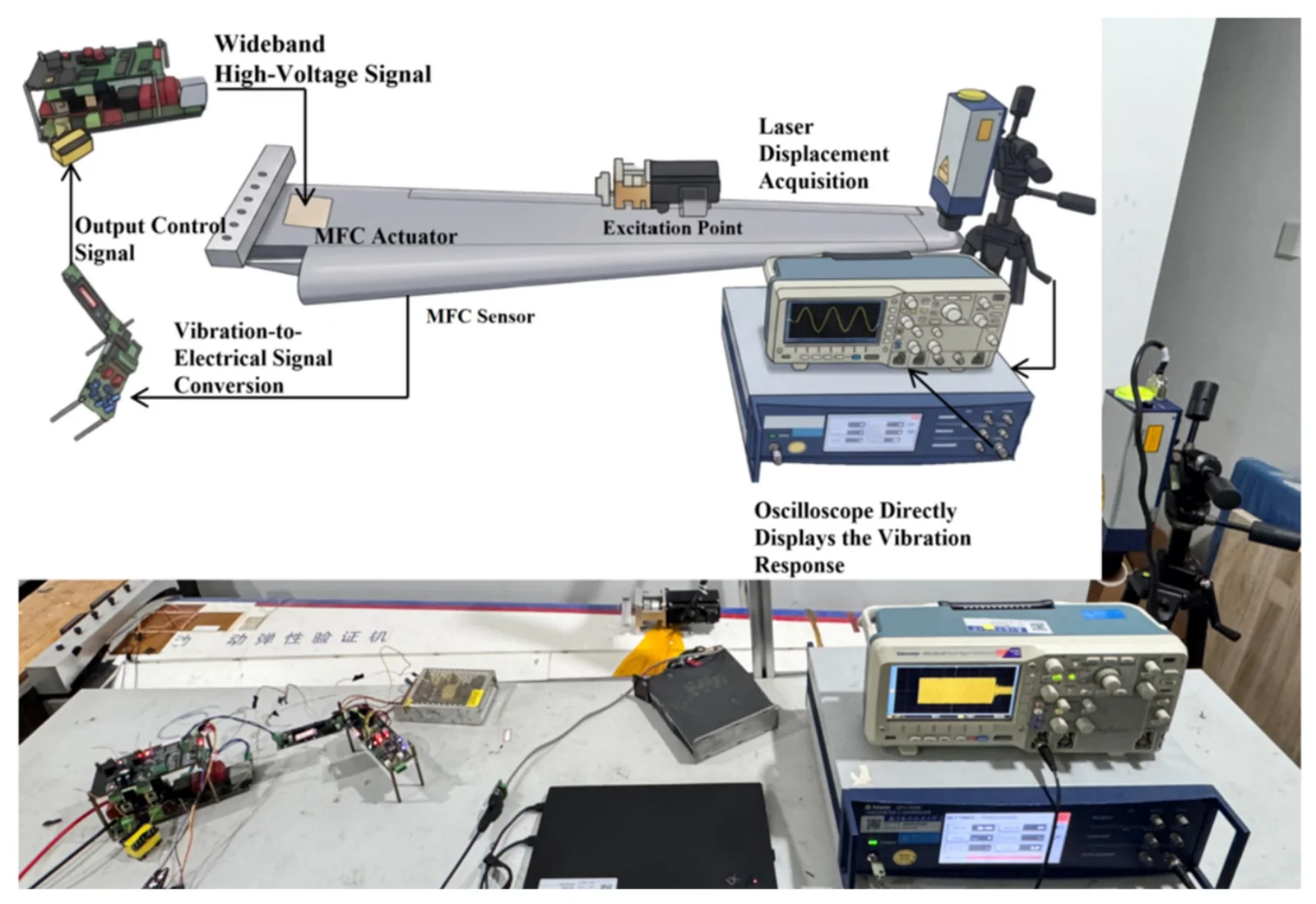
Experimental setup for active vibration suppression of a high-aspect-ratio wing.

Figure 7 presents the experimental setup for active control, specifically designed to perform active vibration-suppression tests on the wing. In the integrated vibration-control experiment, actual (MFC) sensing and actuation (M-8557-P1, CoreMorrow Ltd., Harbin, China) elements were used. The MFC actuator was bonded to the front side of the elastic wing near the root, while the MFC sensor was attached to the mid-span on the rear side of the wing. The excitation frequency was selected within the first bending natural-frequency range of the wing. The dominant structural resonance occurred at approximately 3.6 Hz, where the vibration response reached its maximum amplitude and represented the core operating condition for the vibration-suppression study. A laser vibrometer was used to measure the displacement before and after control. After the digital control module processed the sensor data, it generated a control voltage for the high-voltage driver, which was then applied to the MFC actuator to achieve vibration suppression.

**Figure 8:**
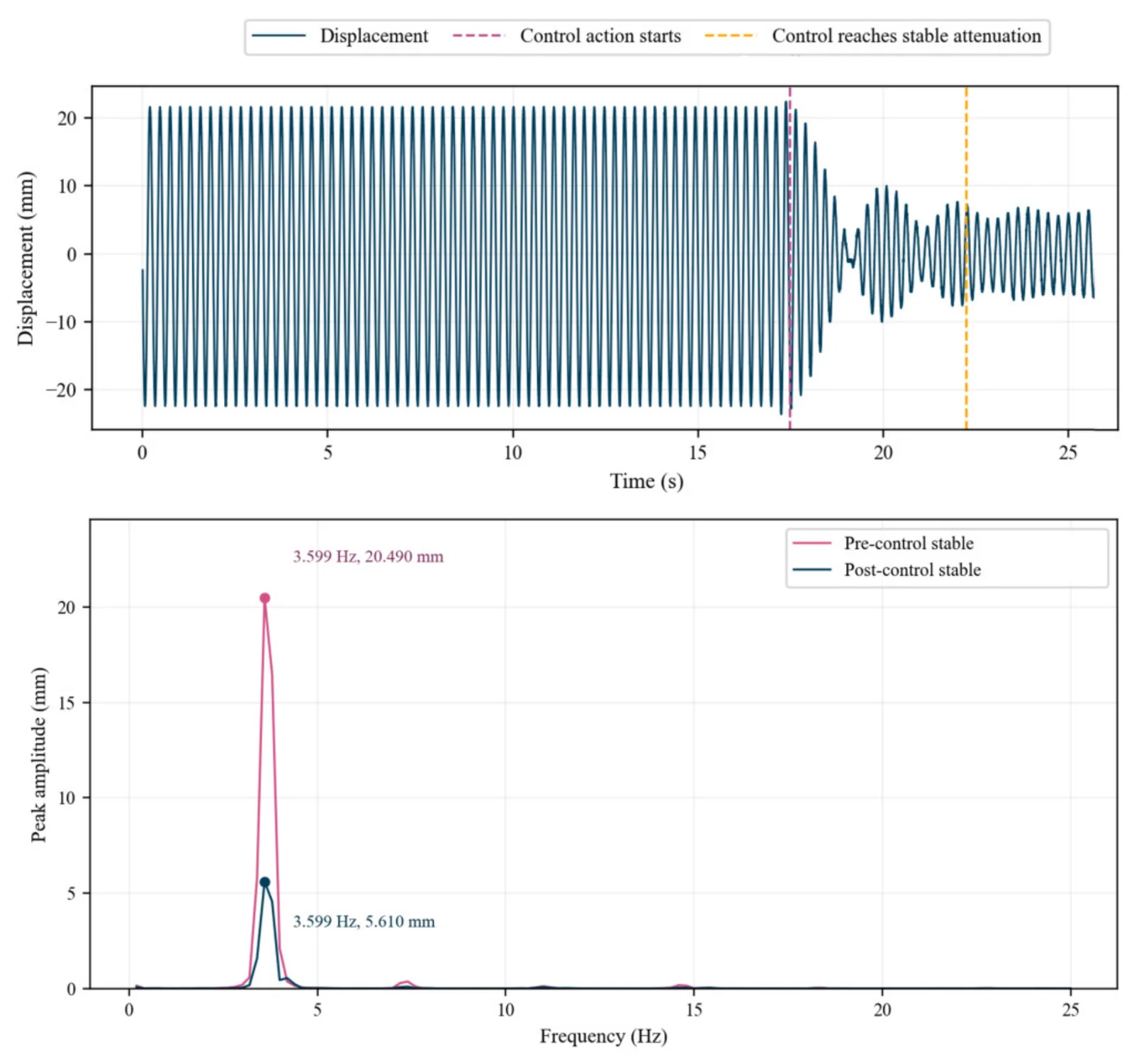
Displacement time history and frequency spectra before vs. after vibration control.

Using the calibration relation 20 mV = 1 mm, the voltage read from CH1 was converted to displacement (mm). The sampling interval was 0.8 ms (sampling rate approximately 1250 Hz). The upper panel in Figure 8 shows the displacement time history. After control was activated, the displacement envelope continuously attenuated and reached a new steady suppression state after approximately 4.770 s.

The root-mean-square displacement was used as a primary indicator of vibration intensity and amplitude. It decreased from 15.567 mm to 4.277 mm, a reduction of 72.52%; the peak-to-peak value decreased from 44.0 mm to 14.0 mm, a reduction of 68.18%. The lower panel in Figure 8 presents the frequency-domain analysis. A windowed Fourier transform using a Hann window and a one-sided amplitude spectrum was applied to the steady-state windows before and after control. The dominant peak frequencies of both datasets were approximately 3.60 Hz, indicating that no dominant-frequency shift was observed. The peak amplitude decreased from 20.49 mm to 5.61 mm, a reduction of 72.62%. The consistency between the time- and frequency-domain indices suggests that the test achieved stable suppression of the dominant resonant component at approximately 3.6 Hz.

## 5. Conclusions

This paper addresses the high-voltage driving requirements of MFCs in active control applications, particularly their asymmetric high-voltage operating range and capacitive-load characteristics, and presents a laboratory prototype of a high-voltage driver. The prototype adopts a voltage–current dual closed-loop structure and a current-tracking PWM control strategy. A real-time embedded control system and active control algorithm based on the STM32G431 microcontroller were developed. In addition, auxiliary signal-conditioning and acquisition modules and an isolation module were designed to integrate MFC piezoelectric sensing, signal acquisition, control computation, and high-voltage driving, thereby forming a high-voltage closed-loop control system for MFC piezoelectric sensing and actuation.

Under the laboratory test conditions reported in this study, step-response and frequency-response tests of high-voltage outputs were conducted on the high-voltage driver prototype. The test results indicate that, under the tested conditions, the prototype exhibits a relatively fast transient response and a certain dynamic driving capability for capacitive loads.

Closed-loop vibration-suppression tests were conducted on a high-aspect-ratio unmanned aerial vehicle wing in the laboratory. Under the tested condition, the dominant vibration frequency was approximately 3.6 Hz. The response reached a steady state about 4.77 s after control was applied. The steady-state root-mean-square displacement decreased from 15.57 mm to 4.28 mm, a reduction of 72.52%. This result demonstrates the basic closed-loop control functionality of the developed prototype system under this representative single-frequency laboratory condition and indicates a certain effectiveness in the active vibration-suppression experiment of the flexible wing. The present work may provide experimental evidence for subsequent research and validation of MFC active vibration-suppression systems.

At present, the system remains a laboratory prototype, and further research is still required before practical engineering applications can be considered. Future work may further address system-level issues, including insulation coordination, shielding, and heat dissipation. On this basis, engineering-oriented verification of output stability, thermal behavior during extended operation, protection performance, and electromagnetic compatibility is still needed to gradually accumulate experimental evidence for system engineering adaptation.

## Figures and Tables

**Table 1:** Step-response results under four bipolar output-voltage levels.

Output Range	($V_{max}$)/V	($V_{pp}$)/kV	Rise Time/μs	Fall Time/μs
(−500) to (+500) V	500	1.00	50	30
(−650) to (+650) V	650	1.30	30	20
(−780) to (+780) V	780	1.56	25	15
(−840) to (+840) V	840	1.68	18	12

## Data Availability

The data that support the findings of this study are available from the corresponding author upon reasonable request.
